# Formulation Development of Albendazole-Loaded Self-Microemulsifying Chewable Tablets to Enhance Dissolution and Bioavailability

**DOI:** 10.3390/pharmaceutics11030134

**Published:** 2019-03-20

**Authors:** Somchai Sawatdee, Apichart Atipairin, Attawadee Sae Yoon, Teerapol Srichana, Narumon Changsan, Tan Suwandecha

**Affiliations:** 1 Drug and Cosmetics Excellence Center, Walailak University, Thasala, Nakhon Si Thammarat 80161, Thailand; attawadee.sa@wu.ac.th; 2 School of Pharmacy, Walailak University, Thasala, Nakhon Si Thammarat 80161, Thailand; 3 Drug Delivery System Excellence Center and Department of Pharmaceutical Technology, Faculty of Pharmaceutical Sciences, Prince of Songkla University, Hat Yai, Songkhla 90112, Thailand; teerapol.s@psu.ac.th; 4 Faculty of Pharmacy, Rangsit University, Pathumtani 12000, Thailand; narumon.c@rsu.ac.th; 5 Department of Pharmacology, Faculty of Sciences, Prince of Songkla University, Hat Yai, Songkhla 90112, Thailand; tan.s@psu.ac.th

**Keywords:** albendazole, self-microemulsion, chewable tablet, dissolution, bioavailability, pharmacokinetics

## Abstract

Albendazole is an anthelmintic agent with poor solubility and absorption. We developed a chewable tablet (200 mg drug equivalent), containing a self-microemulsifying drug delivery system (SMEDDS), with oral disintegrating properties. The emulsion was developed using sesame and soybean oils along with surfactant/co-surfactants, and the tablets were prepared by wet granulation using superdisintegrants and adsorbents. Infra-red (IR) spectral studies revealed no interaction between the drug and excipients, and all physical and chemical parameters were within acceptable limits. Stability studies for the formulation indicated no significant change over time. An in vitro release study indicated 100% drug release within 30 min, and in vivo plasma concentrations indicated that the area under the curve (AUC) of albendazole in rats administered SMEDDS chewable tablets was significantly higher than in those administered commercial tablets or powder (*p*-value < 0.05). The systemic bioavailability of albendazole achieved through the SMEDDS tablets was 1.3 times higher than that achieved by the administration of comparable quantities of albendazole commercial tablets. This was due to the higher dissolution of albendazole SMEDDS in the chewable tablets. We conclude that the SMEDDS chewable formulation can be used to improve the dissolution and systemic availability of poorly water-soluble drugs.

## 1. Introduction

**Figure 1:**
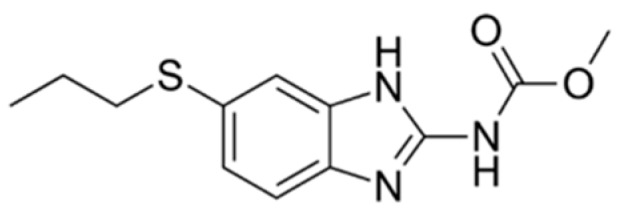
Chemical structure of albendazole.

Albendazole, or methyl(6-(propylthio)-1*H*-benzoimidazol-2-yl) carbamate (Figure 1), a benzimidazole derivative, is a broad-spectrum anthelmintic agent with good efficacy in the treatment of echinococcosis, hydatid cysts, and neurocysticercosis caused by nematodes and cestodes [1,2,3]. It is poorly soluble, with an aqueous solubility of 0.2 μg/mL at 25 °C, 1 μg/mL at pH 6.0, and a log *p* value of 3.5. It has weak basic properties (pK_a1_ = 2.68 and pK_a2_ = 11.83) [4,5]. Albendazole falls into the biopharmaceutical classification system (BCS) class II category with a high permeability and low solubility. Because of its low aqueous solubility, it is poorly and erratically absorbed following oral administration. Following oral administration in rats, 20–30% is absorbed, and in humans, less than 5% is absorbed [1,2,4,6].

To Improve the drug dissolution rate, several techniques have been developed and investigated such as particle size reduction, solid dispersion, inclusion complex formation, use of solubilizing agents, enhancement by surfactant systems, prodrug strategies and drug derivatization, lipid-based formulations, orally disintegrating tablets, self-emulsifying drug delivery system (SEDDS), and self-microemulsifying drug delivery system (SMEDDS) [7,8,9]. SMEDDS is a mixture of oils, surfactants and/or co-surfactant, one or more hydrophilic solvents and co-solvent [9,10,11]. Poorly water-soluble drugs can be dissolved in SMEDDS for oral administration. Upon contact with the aqueous phase of the GI tract, the digestive motility of the stomach and the intestine provide the necessary agitation for the spontaneous and fine dispersion of the SMEDDS formulations because the free energy required to form the emulsion is either low and positive or negative [12]. Although albendazole has been developed as SEDDS [4] and SMEDDS [13] to enhance the dissolution and bioavailability of drugs, liquid dosage forms are inconvenient to carry and difficult to administer as compared to solid dosage forms. In addition, solid pharmaceutical preparations are more stable than liquid preparations and their portability is convenient during the manufacturing process [14]. In fact, SMEDDS does not contain water in their composition, which enhances their chemical and physical stability. The major disadvantage of conventional SMEDDS is the high manufacturing cost as they have to be filled in soft gelatin capsules and they can interact with the shell components of the capsule in the SMEDDS. In addition, precipitation of either active ingredient and/or oil constituents can also be influenced by storage temperature [14,15]. Therefore, attention has been given to transform liquid into solid SMEDDS by several techniques such as spray drying, spray cooling, super critical fluid technology, and using adsorption carriers [14,16]. Colloidal silica, a successful inexpensive hydrophobic carrier, requires common laboratory instruments to formulate as a vehicle for the preparation of solid SMEDDS [16,17]. Carriers with absorbed SMEDDS are then compressed into tablets by wet granulation or direct compression methods [18,19]. However, they are difficult to formulate as tablets due to the high content of surfactant and oil in the formulation. There are no reports on the preparation of albendazole SMEDDS powder or tablets. Commercial albendazole tablets are available as chewable tablet dosage form (Zentel^TM^) in order to achieve rapid drug disintegration. The combination of albendazole SMEDDS with fast disintegrating strategies and their preparation as chewable tablets, similar to the market brand, are important for enhancing drug disintegration and improving dissolution of albendazole, thereby increasing bioavailability.

In this study, we developed a new self-microemulsifying formulation of albendazole combining an orally disintegrating system as a chewable tablet to enhance the dissolution rate of albendazole. We investigated the oral bioavailability of this formulation in rats in comparison with conventional albendazole tablets and powder.

## 2. Materials and Methods

### 2.1. Solubility Studies

An albendazole solubility experiment was carried out according to previous report [4]. Stock solution of albendazole 500 μg/mL in dimethyl sulfoxide (DMSO) was spike in to various oils, surfactants, and co-surfactants by using a 96-well plate format. The 96-well plate was shaken for 2 h and then centrifuged at 4000 rpm at 37 °C on a Sigma centrifuge (Sigma Laborzentrifugen GmbH, Osterode am Harz, Germany) for 10 min. The supernatant was used for high-performance liquid chromatography (HPLC) analysis according to method described in following section.

### 2.2. Construction of Pseudo-Ternary Phase Diagram

Pseudo-ternary phase diagrams were used for the selection of microemulsion area using water titration method. The pseudo-ternary phase diagrams consisting of water, oil, surfactant/co-surfactant mixture of different hydrophilic-lipophilic balance (HLB) values were constructed at room temperature (25 ± 5 °C). The oils employed were sesame oil (Namsiang Co. Ltd., Bangkok, Thailand), soybean oil (Thanakorn Vegetable Oil Products Co., Ltd., Samutprakan, Thailand), Captex 300 Low C6 and Capmul PG8 (medium-chain triglycerides and propylene glycol monocaprylate, respectively, both from Abitec Corporation, Columbus, OH, USA) and Labrafac Lipofile WL1349 (Gattefosseé, SA, France). Surfactant and co-surfactant were Tween80 (Namsiang Co. Ltd., Bangkok, Thailand), Solutol HS 15 (poly-oxyethylene esters of 12-hydroxystearic acid; Sigma-Aldrich, St. Louis, MO, USA), Cremophor RH40 (PEG 400 hydrogenated castor oil, BASF, Washington, NJ, USA) and propylene glycol (S. Tong Chemical Co., Ltd., Nonthaburi, Thailand). The ratio of surfactant and co-surfactant was fixed at 1:1, 1:2, 1:3, 3:1 and 2:1 on the mass ratio. The mixtures of surfactant and co-surfactant with water were prepared at ratios of 10:0, 9:1, 8:2, 7:3, 6:4, 5:5, 4:6, 3:7, 2:8, 1:9, and 0:10 (*w*/*w*). Each mixture of oil and surfactant/co-surfactant was titrated with water and visually observed for phase clarity and flowability. The titration endpoint was defined as the point where the mixture became turbid and phase separation was observed. The resulting mixtures were identified as microemulsions when they appear monophasic, transparent or translucent and easily flowable liquid with low viscosity. The microemulsion regions are indicated on the ternary graph.

### 2.3. Preparation of Albendazole Self-Microemulsifying Drug Delivery System (SMEDDS)

After a series of self-microemulsion formulations was selected from the ternary diagram of surfactants, co-surfactants, and oils, the solubility of albendazole was carried out by HPLC assay. The microemulsion system made with the highest solubility of albendazole was selected to prepare albendazole SMEDDS. An albendazole SMEDDS were prepared as a stock solution at a concentration 0.4% *w*/*w* and stored at room temperature until further use. Briefly, albendazole was accurately weighed, then placed in a beaker, dispersed into an oil phase, and heated at 40–50 °C under vortex. Surfactant and co-surfactant were mixed together in a separate test tube and mixed well under vortex, then heated to 60 °C to mix properly. The drug-containing oil phase was transferred into the surfactant and co-surfactant mixture under continuous mixing, vortexed and heated at 50 °C in a sonicator until albendazole was completely dissolved.

### 2.4. Emulsion Droplet Size Measurement

The particle size measurement was carried out by using a Malvern Zetasizer (Worcestershire, UK) equipped with 2000 Hydro MU at 25 °C. The particle size measurement was in a range 0f 0.02–2000 μm. Each microemulsion was aliquot of 500 μL and diluted to 250 mL with Milli-Q water in a beaker using a magnetic stirrer. The resultant emulsion was analysis and particle size was calculated based on volume size distribution.

### 2.5. Formulation Development of Albendazole SMEDDS Chewable Tablets

Albendazole SMEDDS chewable tablets were prepared using an optimized microemulsion system based on the procedures described above, with a target dose of 200 mg. Albendazole was dissolved in the microemulsion system at a concentration of about 0.4 mg/mL, corresponding to an albendazole dose of 40 mg. 160 mg of albendazole powder was further added to prepare the SMEDDS chewable tablets.

To prepare 1000 tablets, albendazole 40 g was dissolved in a microemulsion system at 100 mL. This was vortex mixed and heated at 50 °C in a sonicator until albendazole dissolved. Albendazole powder and all excipients were passed through a No. 40 sieve before use. Albendazole powder 160 g was mixed with dried lactose monohydrate (P.C. Drug Center Co., Ltd., Bangkok, Thailand), maltodextrin (Brentag Ingredients Public Co. Ltd., Bangkok, Thailand), milk powder and cocoa powder (Cocoa Dutch^®^ 100% instant cocoa), sucrose (Mitr Phol^®^ pure refined sugar), and sodium starch glycolate (P.C. Drug Center Co., Ltd., Bangkok, Thailand) until homogeneous. Albendazole microemulsion was then absorbed in colloidal silicon dioxide (P.C. Drug Center Co., Ltd., Bangkok, Thailand), maltodextrin, and mannitol (P.C. Drug Center Co., Ltd., Bangkok, Thailand) (1:1:1 ratio by weight) and added to albendazole mixture powders using a planetary kneader at a mixing speed of 100 rpm until a homogeneous paste formed. This was dried in a vacuum oven at 70 °C for 12 h to keep the moisture content below 2%. The dried granules were then passed through a No. 14 sieve. Sodium starch glycolate was added as an extragranular disintegrant and vanilla powder and lactose monohydrate were added as diluents (all obtained from P.C. Drug Center Co., Ltd., Bangkok, Thailand) to maintain an equivalent weight.

Magnesium stearate (P.C. Drug Center Co., Ltd., Bangkok, Thailand) was used as a tablet lubricant and was passed through a No. 60 sieve before use. Albendazole SMEDDS granules were blended with sodium starch glycolate used as a superdisintegrant for 5 min in a plastic bottle, then further blended with sieved magnesium stearate (P.C. Drug Center Co., Ltd., Bangkok, Thailand) for 3 min in the same bottle. The final mixtures were compressed into 700 mg tablets using a single punch tableting machine (small tablet press machine Model SP-KR, Charatchai machinery, Thailand) with a round punch and a die diameter of 12 mm. The compression forces (50 kN) were kept constant in order to compare other properties.

**Figure 2:**
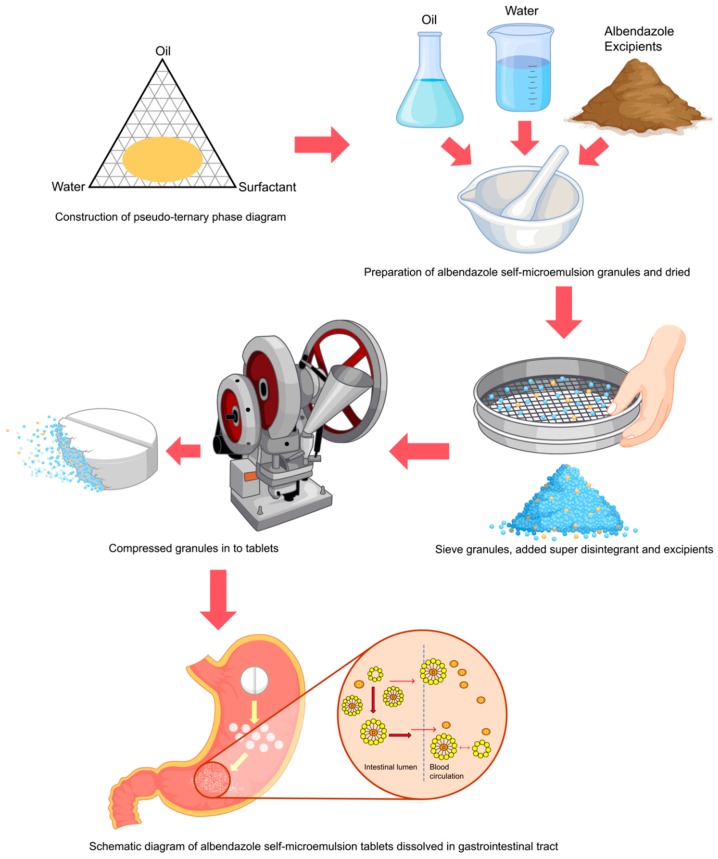
Schematic diagram of the method preparation of albendazole self-microemulsifying drug delivery system (SMEDDS) chewable tablets with a proposed dissolution mechanism enhancing bioavailability.

Physical mixing formulation without the granulating process (F3) was prepared in a similar manner to serve as controls. Briefly, albendazole and all excipients were mixed and compressed powder into a tablet as F3. This formulation was prepared by direct compression process without granulating solvent. Formulation without the albendazole microemulsion (F4) were prepared by mixing albendazole 200 mg/tablet with other excipients excluding the microemulsion system, with purified water added as a granulating solvent and mixed to obtain wet mass. Lactose monohydrate was used in this formulation in place of the microemulsion system. The damp mass was sieved and dried in a hot air oven (70 °C) and passed through a No. 14 sieve, mixed with an extragranular excipient, and compressed into tablets. A schematic diagram of this process is shown in Figure 2, and the details of each formula are given in Table 1.

### 2.6. Analysis of Albendazole by High-Performance Liquid Chromatography (HPLC)

The concentrations of albendazole and albendazole sulfoxide in the formulations and in plasma samples were determined using HPLC as previously reported with a modification [4,20,21,22]. Method validation (specificity, linearity, precision, accuracy, limit of detection (LOD), and limit of quantitation (LOQ)) was performed before analysis. In addition, the system’s suitability parameter was also calculated. The HPLC instrument (Ultimate 3000, Thermo Fisher Scientific, Dionex Corporation, Sunnyvale, CA, USA) equipped with quaternary pump, degasser, a sample loop with an injection volume of 20 μL, and an autosampler. Data was recorded using Chromeleon 7 software. Separations were performed on a 250 mm long × 4.6 mm internal diameter reversed-phase stainless steel column (Inertsil^®^ ODS-3, GL Sciences Inc., Japan) filled with 5 μm octadecylsilane and maintained at 25 °C. The mobile phase consisted of a degassed mixture of hexane and ethanol in a ratio of 89:11 by volume at ambient temperature. The flow rate was maintained at 1.0 mL/min, and separation was monitored by ultraviolet (UV) detection at a wavelength of 291 nm. The calibration curve was found to be linear in a range of 0.02–4 µg/mL.

### 2.7. Powder X-ray Diffraction (PXRD)

Diffractograms of F1–F4 were developed using a powder X-ray diffractometer (Rigaku RU200, Rigaku Corp., Tokyo, Japan). The measuring conditions were as follows: graphite-monochromated Cu Kα radiation; voltage 40 kV, 300 mA and angle speed of 4 °C/min over the range of 5–45°.

### 2.8. Fourier Transform Infrared Spectroscopy (FT-IR)

A small amount of albendazole raw material, albendazole SMEDDS chewable tablets (F1, F2), control tablet formulation (F3, F4) and excipients were grinding mixed into KBr pellets in a small mortar and pestle. The sample with KBr mixture paste was compressed into tablets using a hydraulic press prior to measurement of the infrared (IR) spectrum at ambient temperature. The functional groups of albendazole, albendazole formulations and excipients were recorded in the frequency range of 4000–400 cm^−1^ using a Fourier transform infrared (FT-IR) spectrophotometer (Perkin Elmer Inc., Waltham, MA, USA).

### 2.9. Physical and Mechanical Properties of Granules and Tablets

#### 2.9.1. Angle of Repose

Granule or powder formulations were each placed in a funnel hung at a fixed height using a burette stand and allowed to fall onto a graph paper, forming a heap. The height and the radius of the heap was measured and the angle of repose was calculated using the formula given in Equations (1) or (2).

(1) $$\tan\;\theta =\;\frac{\mathrm{height}\;\mathrm{of}\;\mathrm{the}\;\mathrm{heap}\;\mathrm{formed}\;(h)}{\mathrm{radius}\;\mathrm{of}\;\mathrm{the}\;\mathrm{heap}\;(r)},$$

or

*θ* = tan^−1^ h/r (2)

#### 2.9.2. Hardness Test

The hardness of the F1–F4 tablets was measured using a PTB311E model (Pharma Test, Hainburg, Germany) and expressed as a mean value standard deviation.

#### 2.9.3. Thickness Test

The dimensions of F1–F4 tablets were measured using a Vernier caliper. Six measurements were taken and expressed as a mean value ± standard deviation.

#### 2.9.4. Friability Test

Ten tablets of each category were accurately weighed and placed in a plastic chambered friability apparatus (Erweka, model TA220, Heusenstamm, Germany) described in USP36 [23]. The chamber was attached to a motor revolving at 25 rpm for 4 min. The tablets were weighed again, and the percentage weight loss (friability) was calculated using the following formula:

(3) $$\mathrm{Friability}=\frac{\mathrm{Initial}\;\mathrm{weight}-\mathrm{Final}\;\mathrm{weight}}{\mathrm{Initial}\;\mathrm{weight}}\times 100$$

#### 2.9.5. Disintegration Test

The disintegration test for the tablets was performed using a disintegration apparatus with discs (Pharma Test, model DIST3, Hainburg, Germany) described in USP36 [23] with six replicates for each tablet group. Tablets were placed individually in each tube in a 900 mL beaker of distilled water maintained at a temperature of 37 ± 2 °C. The average values of the disintegration time and standard deviations were calculated.

### 2.10. Content of Albendazole SMEDDS Chewable Tablets

Ten tablets of selected formulation were weighed and grounded to a fine powder, and a quantity equivalent to 200 mg albendazole (700 mg of powder) was introduced into a 100 mL volumetric flask and diluted with the mobile phase. The solution obtained was sonicated for 15 min and filtered through a 0.45 μm nylon membrane filter. The filtrate was suitably diluted with the mobile phase prior to HPLC analysis as previously described. The mean percentage of the drug content was determined based on three replicates.

### 2.11. In Vitro Dissolution Studies

A United State Pharmacopoeia dissolution apparatus II (Varian, Vankel VK7010, Palo Alto, CA, USA) with paddle rotation speed of 50 rpm was used to monitor the in vitro dissolution profiles of albendazole. The dissolution values of albendazole self-microemulsion chewable tablets (F1 and F2) were compared to the albendazole control formulation by physical mixing method (F3), conventional wet granulation without SMEDDS excipient (F4) and a commercial product which was purchased from a drug store in Thailand. The dissolution profile was performed in 900 mL 0.1 M HCl as the dissolution medium described in several research works at 37 ± 0.5 °C [23,24,25,26]. During the study, 5 mL aliquots were taken at predetermined time intervals from the dissolution medium and 5 mL of fresh medium were replaced. Samples were withdrawn from the dissolution vessels at 0, 0.25, 0.5, 0.75, 1, 1.5, 2, 2.5, 3, 3.5, 4, 4.5, 5, 10, 20, and 30 min and passed through a 0.45 μm nylon membrane filter prior to analysis. The amount of albendazole was determined using HPLC as described above. The dissolution experiments were carried out in triplicate.

### 2.12. Stability Studies

Selected formulations of the albendazole SMEDDS chewable tablets were packed in Alu-PVC blister packaging covered with aluminum foil, in order to block moisture and light, and kept under accelerated conditions (40 °C/75% relative humidity (RH)), long-term stability conditions (30 °C/75% RH), and at room temperature with ambient relative humidity following the Association of Southeast Asian Nations Guidelines the Stability of Drug Products according to climatic zone IVb [27,28]. Tablets were evaluated for appearance, disintegration time, and drug content over periods of 1, 3, and 6 months.

### 2.13. Animals

Male Wistar rats were purchased from the National Laboratory Animal Center, Mahidol University, Thailand. Animals were fed until weighting about 250–300 g before used. All animals had free access to pelleted food and tap water *ad libitum* prior to the experiments, and were housed in clean polypropylene or corrugated paper cages. Temperature was maintained at controlled room temperature (25 ± 2 °C) and humidity of 50–60% with a 12 h light and dark cycle throughout the experiment. These experimental procedures were approved by the Animal Ethical Committee, Walailak University, Nakhon Si Thammarat, Thailand (approval no. 004/2559) before performed the experiment.

### 2.14. Drug Administration and Sampling

Overnight fasted rats were divided into three groups with five rats in each. Albendazole SMEDDS chewable tablets, commercial albendazole tablets (Zentel^TM^), or albendazole raw material were administered orally as a single dose (p.o.) using a gastric gavage tube to rats in each group. The selected formulation tablets (F2) or Zentel^TM^ tablets were crushed to a fine powder by mortar and pestle. The fine tablets powder (F2 or Zentel^TM^) and albendazole raw material were weight equivalent to 50 mg per kg of each animal weight. The drug powders were each dispersed in 2 mL of distilled water and mixed homogeneously prior to oral administration. Blood samples (0.5 mL) were collected via the tail artery at 0, 15, 30, 60, 90, 120, 180, and 240 min after the oral administration of albendazole. The samples were immediately transferred to a heparinized microcentrifuge tube and centrifuged at 4000 *g* for 20 min at 4 °C. Plasma samples were removed to Eppendorf tubes for further use. During the study, the animals received water ad libitum.

### 2.15. Preparation of Samples

Each 400 µL of separated plasma sample was immediately mixed with 2 mL methanol in a vortex mixer for 15 s and centrifuged at 2000 rpm for 5 min. The supernatant was collected and stored at −70 °C prior to use. The procedures used for sample preparation and handling were done within 24 h of blood sample collection. The supernatant was transferred to another Eppendorf tube for HPLC analysis [20].

### 2.16. Pharmacokinetics and Statistical Analysis of Data

After treatment in each animal, the albendazole sulfoxide concentration versus time curves obtained from individual animals were fitted using WinNonlin software version 5.2 (Pharsight Corp, Mountain View, Sunnyvale, CA, USA) and reported as mean ± standard deviation (SD). The pharmacokinetic parameters for each animal were analyzed via non-compartmental model analysis for three formulations (albendazole SMEDDS chewable tablets, albendazole tablets commercial product, and albendazole powder). The pharmacokinetic parameters including the area under the concentration-time curve (AUC_0–∞_), maximum concentration (*C*_max_), time to reach maximum concentration (*T*_max_) and half-life (*t*_1/2_) were determined by trapezoidal rule. The relative bioavailability (F) was calculated according to the following equation:

(4) $$F=\frac{\mathrm{AUC}\;(\mathrm{test}\;\mathrm{formulation})}{\mathrm{AUC}\;(\mathrm{reference})}$$

where AUC of the reference is the AUC of the albendazole commercial product group and AUC test formulation is the AUC of albendazole SMEDDS chewable tablet or albendazole powder.

The pharmacokinetic parameters were reported as mean ± SD. Pharmacokinetic parameters were statistically compared using a one-way analysis of variance (ANOVA). Mean values were considered significantly different at *p* < 0.05.

## 3. Results and Discussion

### 3.1. Construction of a Pseudo-Ternary Phase Diagram

SMEDDS are a mixture of oil, surfactant, and co-surfactant with a drug dissolved in the system to improve the absorption of poorly water-soluble drugs. These systems form a microemulsion when exposed to an aqueous phase in the GI tract under mild agitation. Therefore, to select the SMEDDS composition for albendazole, its solubility in various vehicles was studied. The results indicated that sesame oil, soybean oil, Capmul PG8, Labrafac Lipophile WL1349, Cremophor RH40, Tween80, and PEG400 were the most effective in developing solvent mixtures because of the high solubility of albendazole (data not shown) in these vehicles. Therefore, we used these ingredients to construct a pseudo-ternary phase diagram.

**Figure 3:**
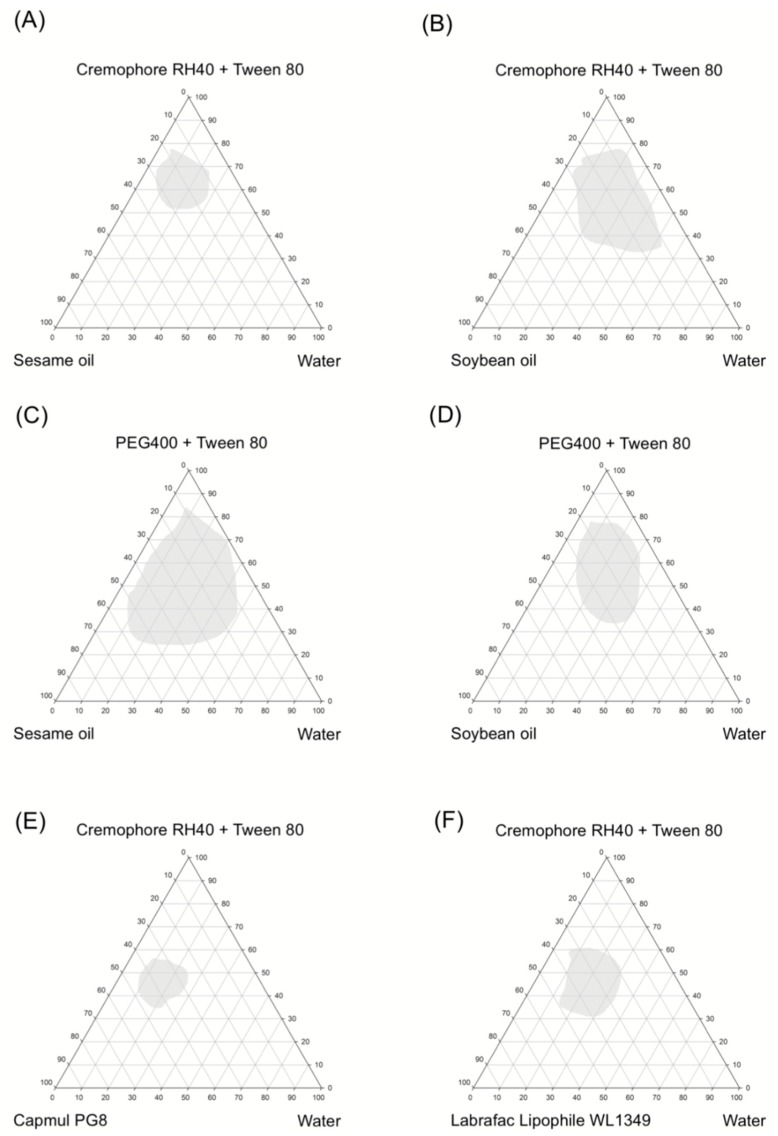
Pseudo-ternary phase diagram of various surfactant, co-surfactant, oil, and water. Microemulsion regions of the ternary plot are indicated in the gray areas.

**Figure 4:**
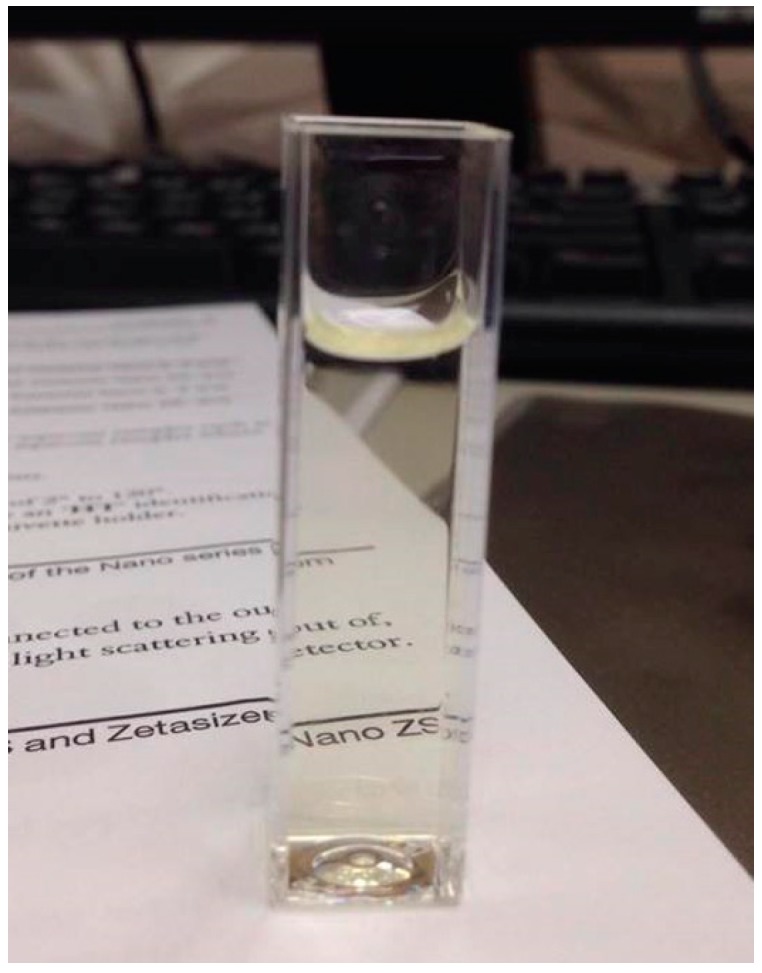
Appearance of microemulsion containing sesame oil, PEG400, Tween80 and water.

The construction of a pseudo-ternary phase diagram in the absence of active ingredient was used to identify the optimized concentrations of oil, surfactant and co-surfactant in the liquid SMEDDS formulation. Their self-emulsification properties were visually observed after SMEDDS were prepared [17,29,30]. Diagrams of oil (sesame oil, soybean oil, Capmul PG8, and Librafac Lipophile WL1349), surfactants and co-surfactants (Cremophor RH40, Tween80, and PEG 400), and water are provided in Figure 3. A large microemulsion region obtained from sesame oil with PEG400 and Tween80, in a 2:1 ratio, in water yielded a clear and transparent solution (Figure 3C). A second large microemulsion region was prepared using soybean oil with Cremophor RH40 and Tween80 in a ratio of 2:1 (Figure 3B). The microemulsion containing 20% sesame oil, 40% PEG400:Tween80 (2:1), and 40% water is shown in Figure 4. Sesame and soybean oils have been developed as self-nanoemulsifying drug-delivery systems due to their solubilizing capability and low toxicity [31,32]. Thus, sesame oil and soybean oil were selected as oil phases for the preparation of albendazole-loaded SMEDDS containing chewable tablets in the next step. Cremophor RH40 (PEG-40 hydrogenated castor oil and HLB 14–16) and PEG400 were used as the surfactant and co-surfactant, respectively. Cremophor RH40 is widely used as an emulsifying and solubilizing agent especially for albendazole [4]. Generally, non-ionic surfactants are considered less toxic than ionic surfactants, and are usually accepted for oral ingestion [33,34]. PEG400 and Tween80 also enhanced drug solubility, and when mixed with Cremophor RH40, yielded a suitable HLB value and viscosity to form a fine microemulsion. Furthermore, PEG400 and Tween80 are powerful solubilizing agents used in several dosage forms [32]. Based on these results, the compositions of sesame oil, soybean oil, PEG 400, Tween80, and Cremophor RH40 were selected for preparing self-microemulsions of albendazole granules.

Based on the pseudo-ternary phase diagram, soybean oil with Cremophor RH40/Tween80 and sesame oil with PEG400/Tween80 in water (Figure 3B,C) showed a large microemulsion region. The z-average particle size of the liquid and solid SMEDDS were assessed. From Figure 3B,C, in the soybean oil or sesame oil/PEG400 or Cremophor RH40/Tween80 system, we observed that the liquid SMEDDS formulation of 20% of soybean oil or sesame oil, 30% Cremophor RH40, and 50% Tween80 showed the smallest z-average diameter (around 150 nm for both systems) (Table 2). Therefore, this composition was used as an optimal liquid SMEDDS. Furthermore, 10% (*w*/*w*) of the drug was entirely dissolved in this formulation and particle size larger than liquid SMEDDS without the drug but these were not significantly different. Moreover, the z-average particle sizes of other liquid SMEDDS system (Figure 3A,D–F) were similar.

The assessment of self-emulsification can be carried out by visually observing the emulsification and droplet formation of the liquid SMEDDS formulations. Spontaneous emulsion formation was not efficient when the volume of surfactant was less than that of the oil in liquid SMEDDS. In the case of sesame oil/PEG400/Tween80 system, the efficiency of emulsification was good when the concentration of the surfactant/co-surfactant was more than 60% *v*/*v* of the liquid SMEDDS formulation. Similarly, in the case of soybean oil/Cremophor RH40/Tween80 system, the efficiency of emulsification was good when the concentration of the surfactant/co-surfactant was more than 70% *v*/*v* of the liquid SMEDDS formulation.

### 3.2. Preparation of Albendazole Self-Microemulsion Chewable Tablets

Solid carriers are mainly divided into either water-soluble carriers or water-insoluble carriers [35,36]. Colloidal silicon dioxide is non-porous silica with hydrophobic properties. It is used as a water-insoluble carrier and approximately 1 g of colloidal silicon dioxide can be used to solidify 1 g of SMEDDS [35,36]. Water-insoluble carriers have high oil-adsorbing capacity and thus minimize the amount required to solidify the SMEDDS, but an incomplete desorption of SMEDDS components can occur because of hydrophobic interactions between the drug and water-insoluble solids [36,37,38]. Mannitol is a non-hygroscopic isomer of sorbitol and also used as a solidifying carrier [36,39,40]. Maltodextrin is a polysaccharide and widely used as a tablet excipient to improve tablet properties such as liquid absorbance and increased porosity with rapidly absorbing capacity [41,42,43]. Only colloidal silicon dioxide is enough for absorbing liquid SMEDDS with albendazole. Thus, in this study, maltodextrin and mannitol were used as tablet excipients while formulating the chewable tablets. However, other absorbents can be for absorption during the granule preparation. In this study, we selected sodium starch glycolate as a disintegrant due to its fast-disintegrating properties. Other ingredients including milk powder, cocoa powder, sugar, and vanilla flavor powder were chosen for their good taste in chewable tablets. In addition, we selected lactose for solidification, because of its popularity as a diluent in commercial products.

**Figure 5:**
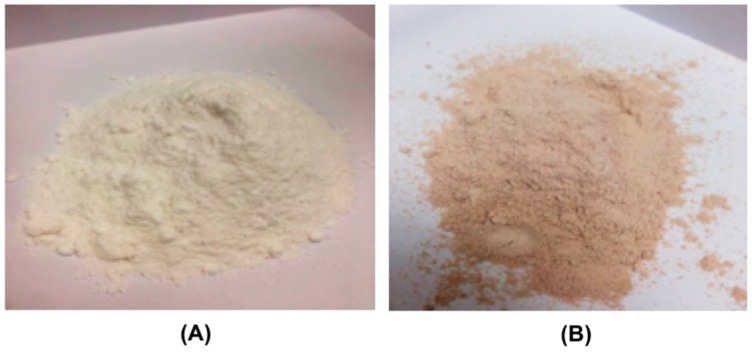
Appearance of albendazole self-microemulsifying chewable tablets: (**A**) microemulsion absorbed from colloidal silica and (**B**) albendazole self-microemulsifying granules.

Since about 40 mg albendazole was dissolved in the self-microemulsion system, albendazole powder was added to the formulation at 160 mg per tablet (Table 3). Figure 5A shows the appearance of the albendazole self-microemulsifying powder after the absorption of colloidal silicon dioxide in it, and Figure 5B depicts the appearance after the absorbed albendazole was formulated as a granule by adding tablet excipients, prior to tableting.

The flow characteristic of the SMEDDS formulations in terms of angle of repose was determined. In all SMEDDS formulations, flow properties increased when incorporating the self-microemulsion system (F4). The values for angle of repose of less than 40° yields a fair powder flow requiring no aid, and less than 35° represents good flow properties [23]. Flow properties based on angle of repose values were observed in the order of F2 (31.1°) > F1 (32.4°) > F3 (33.2°) > F4 (34.1°) as shown in Table 3, indicating good free-flowing properties in all samples [23].

### 3.3. Physical and Chemical Characteristics of Albendazole Self-Microemulsion Chewable Granules and Tablets

**Figure 6:**
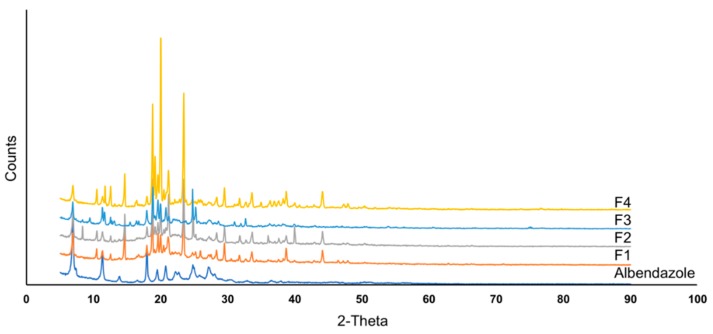
X-ray diffractogram of albendazole and albendazole self-microemulsifying chewable tablets formulation F1–F4.

Albendazole raw material, used as a model drug in this study, is a crystalline solid with irregular shape [26]. Powder X-ray diffraction (PXRD) was performed to identify the crystalline state of albendazole raw material and albendazole self-microemulsifying chewable tablets. The X-ray diffractogram of albendazole in the formulation is shown in Figure 6. Albendazole raw material showed numerous sharp and intense peaks at diffraction angles of 7.18°, 11.28°, 17.93°, 19.48°, 20.78°, 25.33°, and 27.68°, indicating its high crystallinity. The XRD patterns of SMEDDS formulations F1–F2 show sharp and intense crystalline peaks, indicating that the physical state of albendazole, in the formulations, remain crystalline in the SMEDDS formulations. In the same results, formulation F3, a physical mixture of albendazole with SMEDDS excipients, shows a crystalline structure, as suggested by the XRD diffraction patterns, similar to the formulations, F1 and F2. The major peaks of these samples are similar to those obtained in the SMEDDS samples, in terms of intensity and position. The last formulation, F4, was prepared by the conventional wet granulation method without SMEDDS excipients. It showed very high intensity diffraction peaks, probably due to the absence of oil and surfactants with partially dissolved albendazole. Albendazole did not transform from crystalline to an amorphous state, as all the formulations used a high portion of pure albendazole raw material.

**Figure 7:**
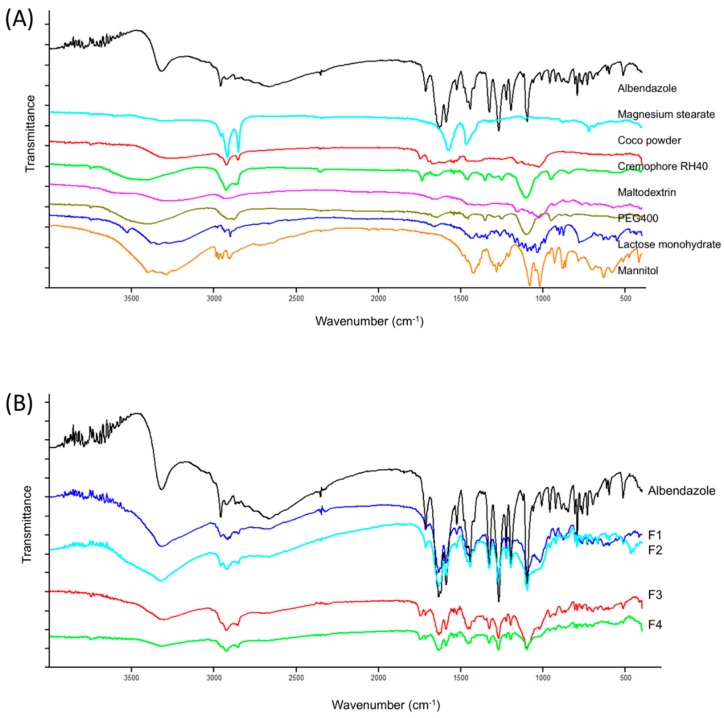
Fourier transfrom infrared (FT-IR) spectrum of (**A**) albendazole and excipients used in the formulation (**B**) albendazole and albendazole SMEDDS chewable tablets formulation F1–F4.

The results obtained from IR studies showed no interaction between the drug and other excipients used in the formulation. FT-IR of albendazole showed intense bands at 1095.6, 1268.8, 1588.7, 1632.1, 1713.8, and 3318.6 cm^−1^, corresponding to the presence of functional groups such as aromatic compounds, carbonyl, alkyl, and amine. The FT-IR of albendazole formulations F1–F4 showed intense bands at the same wave number, indicating no change in the functional groups confirming undisturbed structure of albendazole, and suggesting that there was no drug-excipient interaction (Figure 7).

F1–F4 formulations were evaluated for all physical parameters such as weight variation, diameter, thickness, hardness, and friability (Table 4). Thickness ranged from 5.64 ± 0.02 mm to 5.72 ± 0.06 mm, due to the different composition of tablets and granules, characteristic of each formulation. Weight variation and friability were found to be within United State Pharmacopoeia (USP) specifications [23]. Percent weight variation was well within the acceptable limit for uncoated tablets, as per USP specifications. Tablets with the greatest hardness show longer disintegration time, and since mechanical integrity is of paramount importance in the successful formulation of tablets, the hardness of tablets was determined. The friability of albendazole SMEDDS was less than 1%, which is acceptable according to USP criteria. None of the 10 tablets tested were outside the range of 85–115% of the dosage claimed on their commercial label. These results indicate that the dosage form had uniform distribution and proper dose of the active ingredient. The disintegration time was less than 3 min for all compositions, indicating that they can be used for formulation as chewable tablets. We also found that hardness had no significant effect on drug release, although low hardness decreased the disintegration time. However, since the friability of the tablets was compromised to avoid breaking or erosion, a narrow range of hardness, between 35–45 N, was selected for the compression of batches. Mean values with standard deviation of all physical parameters and drug content for all formulations are shown in Table 3. Drug content of albendazole-loaded SMEDDS preparations are listed in Table 3. In general, the drug content of active ingredients was set within the limit of 90–110% of the labeled claim [23]. All SMEDDS formulations showed drug content in the range of 100–101%, indicating that the albendazole-containing SMEDDS was sufficiently adsorbed onto the solid carriers, whilst drug content was constant, indicating that the manufacturing process did not destroy albendazole during preparation.

### 3.4. In Vitro Dissolution of Albendazole SMEDDS Chewable Tablets

**Figure 8:**
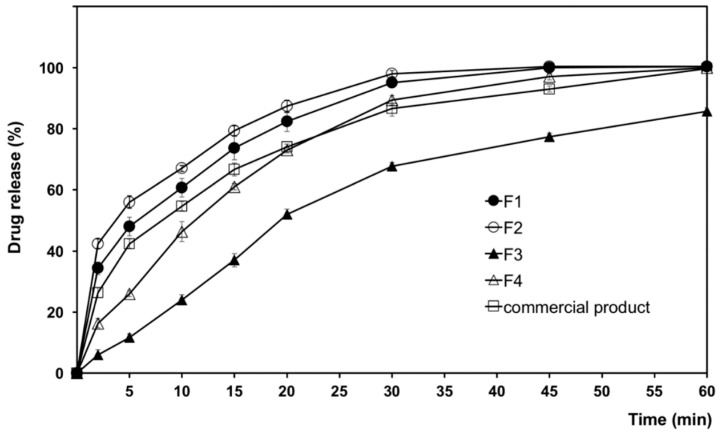
Dissolution profiles of albendazole SMEDDS chewable tablets (F1–F4) and a commercial albendazole tablet in 0.1 N HCl medium pH 1.2.

The cumulative percent of drug dissolved, as a function of time, from albendazole self-microemulsion chewable tablets is illustrated in Figure 8. Dissolution from various samples of F1–F4 was observed in a simulated gastric medium (0.1 N HCl medium, pH 1.2). All the prepared albendazole self-microemulsion chewable tablets (F1 and F2) yielded significantly higher dissolution than the market product, within the 60-min timeframe. The commercial product in this study used Zentel^TM^ (Glaxosmithkline Pharmaceuticals Ltd., Brentford, UK). The composition of Zentel^TM^ consists of lactose, microcrystalline cellulose, maize starch, croscarmellose sodium, povidone, sodium lauryl sulphate, sunset yellow lake, sodium saccharin, magnesium stearate, orange flavor, vanilla flavor, and passion fruit flavor [44]. We believe that sodium lauryl sulphate, as a surfactant in the commercial products, may enhance dissolution of albendazole that higher than formulation F3 and F4. Although formulation F3 consists of ingredients similar to the formulation F2, it is prepared by a physical mixing method. Albendazole in F3 does not dissolve to form a microemulsion before the formulation of the tablets.

The percentage of drug dissolved from test tablets in 60 min can be arranged in descending order as follows: F2 > F1 > market product > F4 > F3, indicating that albendazole self-microemulsion chewable tablets enhanced the dissolution of albendazole. Complete dissolution was achieved from F3 in 30 min. Decrease in the dissolution rate in case of F4 as the formulation did not contain a microemulsion system. In contrast, dissolution of formulation F3 decreased due to the high content of oil, which was not formulated as a self-microemulsion system.

Albendazole dissolution significantly increased to over 20% for the same time period as compared with the formulation without a self-microemulsion system. There was a significant increase in albendazole dissolution in the SMEDDS formulations as compared to the commercial product. The dissolution rates of F2 was similar to F1, due to the complete solubilization of the albendazole in the microemulsion system. SMEDDS improved the drug release rate as compared to the conventional albendazole tablets (F4) or commercial tablets because the free energy required to form a microemulsion is very low and the spontaneous formation of an interface between water and oil droplets is possible [29,45].

Both self-microemulsion systems F1 and F2 showed high dissolution rates (>90% in simulated gastric medium, pH 1.2 for 1 h). F2 was selected for further in vivo pharmacokinetics studies, since it dissolved somewhat faster, and due to its overall superior performance. Therefore, it was subjected to further in vivo studies for comparison with the commercial compressed chewable tablets.

### 3.5. Stability Studies

After six months at accelerated storage at 40 °C/75% RH, storage at 30 °C/75% RH, or storage at room temperature and at ambient humidity, there were few differences in appearance, disintegration time, friability and drug content before and after the storage period (Table 5). At 30 ± 2 °C/75 ± 5% relative humidity, hardness was slightly increased, likely due to the absorbed moisture. This indicates that the formulation was fairly stable at both storage conditions.

The water contents were calculated according to the following equation:

water content (%) = [*W*_t_/*W*_0_] × 100 (5)

where, *W*_0_ and *W*_t_ are the water content in each sample at time 0 and time t, respectively.

Albendazole self-microemulsion chewable tablet, F2, showed no significant drug loss, suggesting that it was the most stable among the formulations tested in this study. Meanwhile, water content slightly increased under all storage conditions, even though there were no significant differences and tablets were not hygroscopic. This is likely due to PVC serving as a poor moisture barrier.

### 3.6. Pharmacokinetic Analysis of Albendazole SMEDDS Chewable Tablets

**Figure 9:**
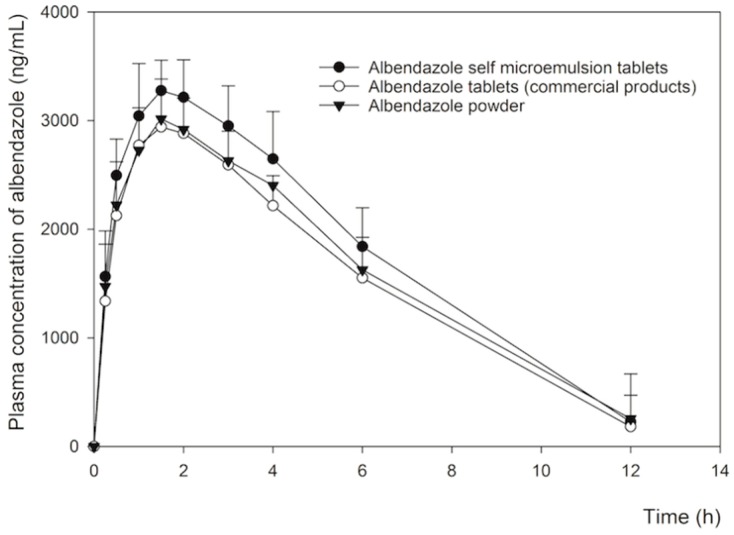
Mean plasma concentration/time profiles of albendazole sulfoxide after oral administration of albendazole at a dose of 50 mg/kg in SMEDDS chewable tablets formulation F2 (solid circles, *n* = 5), albendazole commercial tablets (open circles, *n* = 5) and albendazole powder (solid triangles, *n* = 5) in rats. Data shown indicate mean ± standard deviation (SD).

Figure 9 shows the change in the mean plasma concentration of albendazole after the oral administration of the F2 albendazole self-microemulsion chewable tablet, albendazole commercial chewable tablets, and albendazole powder, at a dose of 50 mg/kg albendazole, in rats. The albendazole commercial tablets and powder yielded a lower total plasma concentration compared to the F2 chewable tablet formulation. One to 12 h after dosing, plasma concentrations of both the commercial tablets and albendazole powder were significantly lower compared with the F2 chewable tablet (*p* < 0.05), likely due to their lower drug solubility and dissolution. Some studies indicate that relatively higher plasma concentrations of albendazole with SMEDDS were due to the increase in solubility and dissolution of albendazole from this system [4,13]. The pharmacokinetic parameters are shown in Table 6. The albendazole SMEDDS formulations showed significantly higher AUC and *C*_max_ compared to the commercial tablets and albendazole powder (*p*-value < 0.05) but the AUC and *C*_max_ of the commercial tablets and albendazole powder showed bioequivalent (*p*-value > 0.05). In particular, the AUC values of albendazole SMEDDS were about 1.3-fold higher than that of the albendazole powder. However, AUC did not differ between albendazole commercial tablets and albendazole powder. Although *C*_max_ values were about 1.2 fold higher as compared to the corresponding values for albendazole powder, *T*_max_ and *t*_1/2_ values of both formulations were not significantly different from the commercial powder. Our results suggest that the enhanced oral bioavailability of albendazole in the SMEDDS formulation led to a marked increase in drug absorption due to its improved solubility and dissolution, supporting the hypothesis illustrated in Figure 1.

## 4. Conclusions

In this study, two formulations of albendazole were developed as SMEDDS based on either sesame oil + PEG400 + Tween80 or soybean oil + Cremophor RH40 + Tween80, in order to increase the solubility, stability, and bioavailability of albendazole. Using this formulation, as a chewable tablet, the dissolution and bioavailability of albendazole improved compared to a commercial formulation and albendazole powder. This tablet was stable under accelerated conditions of 40 °C and 75% RH, long-term conditions at 30 °C and 75% RH, and room temperature with ambient humidity for six months.

## Figures and Tables

**Table 1:** Composition of albendazole self-microemulsion chewable tablet formulations.

Ingredients (mg/tablet)	Formulation Codes			
	F1	F2	F3	F4
Albendazole (in microemulsion)	40	40	-	-
Soybean oil	-	34	34	-
Sesame oil	24	-	-	-
Cremophor RH40	-	33	33	-
Polyethylene glycol (PEG) 400	38	-	-	
Tween80	38	33	33	-
Albendazole (as powder)	160	160	200	200
Maltodextrin	70	70	70	70
Mannitol	70	70	70	70
Colloidal silicon dioxide	70	70	70	70
Lactose monohydrate	20	20	20	120
Sucrose	25	25	25	25
Sodium starch glycolate	28	28	28	28
Milk powder	60	60	60	60
Coco powder	50	50	50	50
Vanilla powder	0.1	0.1	0.1	0.1
Magnesium stearate	7	7	7	7
Purified water *	0.2	0.2	-	0.2
Total weight	700	700	700	700

* evaporated during manufacturing.

**Table 2:** Particle size of liquid SMEDDS with and without 10% albendazole (mean ± SD, *n* = 3).

Liquid SMEDDS	Particle Size (nm)	Polydispersity index (PDI)
20% Sesame oil 30% PEG400 50% Tween80 without albendazole	152.5 ± 10.1	0.234 ± 0.005
20% Sesame oil 30% PEG400 50% Tween80 with 10% albendazole dissolved	168.9 ± 10.3	0.239 ± 0.004
20% Soybean oil 30% Cremophor RH40 50% Tween80 without albendazole	149.9 ± 12.3	0.754 ± 0.012
20% Soybean oil 30% Cremophor RH40 50% Tween80 with 10% albendazole dissolved	159.9 ± 19.1	0.139 ± 0.014

**Table 3:** Solubility of albendazole in selected self-microemulsifying formulations.

Composition	Formulation (%)					
	A	B	C	D	E	F
Sesame oil	34	-	24	-	-	-
Soybean oil	-	34	-	32	-	-
Capmul PG8	-	-	-	-	30	-
Labrafac Lipophile WL1349	-	-	-	-	-	35
Cremophor RH40	33	33	-	45	27	44
Tween80	33	33	38	23	43	21
PEG400	-	-	38	-	-	-
Solubility of albendazole (mg/mL)	0.38 ± 0.02	0.41 ± 0.01	0.43 ± 0.02	0.39 ± 0.02	0.16 ± 0.04	0.28 ± 0.17

**Table 4:** Properties and assay results for the albendazole SMEDDS chewable granules and tablets (F1–F4) (mean ± SD, *n* = 10).

Formulation	Angle of Repose (°)	Thickness (mm)	Hardness (N)	Friability (%)	Disintegration Time (min)	%LA
F1	32.4 ± 0.02	5.65 ± 0.05	38.2 ± 11.8	0.45 ± 0.21	<3	101.21 ± 0.23
F2	31.1 ± 0.03	5.64 ± 0.02	37.2 ± 7.8	0.23 ± 0.11	<3	100.46 ± 1.11
F3	33.2 ± 0.01	5.71 ± 0.04	40.2 ± 4.9	0.16 ± 0.57	<3	100.15 ± 0.59
F4	34.1 ± 0.03	5.72 ± 0.06	41.2 ± 2.0	0.89 ± 0.28	<3	101.05 ± 0.03

**Table 5:** Of the albendazole self-microemulsifying chewable tablet (F2) stored at 30 °C/75% relative humidity (RH) and 40 °C/75% RH (mean ± SD, *n* = 10).

Storage Conditions	Test	Storage Period		
		1 Month	3 Months	6 Months
Room temperature	Appearance	Light brown, round, flat tablet		
	Hardness (N)	38.2 ± 10.8	37.2 ± 4.8	38.2 ± 6.9
	Disintegration time (min)	<3	<3	<3
	Friability (%)	<1	<1	<1
	Water content (%)	3.32 ± 0.13	3.45 ± 0.82	3.51 ± 0.88
	Drug content (%)	100.16 ± 0.64	99.26 ± 1.67	100.92 ± 1.32
30 °C/75% RH	Appearance	Light brown, round, flat tablet		
	Hardness (N)	37.2 ± 11.8	38.2 ± 4.9	41.2 ± 2.0
	Disintegration time (min)	<3	<3	<3
	Friability (%)	<1	<1	<1
	Water content (%)	3.12 ± 0.53	3.65 ± 1.02	4.01 ± 1.12
	Drug content (%)	101.03 ± 1.14	101.26 ± 0.87	101.39 ± 0.23
40 °C/75% RH	Appearance	Light brown, round, flat tablet		
	Hardness (N)	38.1 ± 1.9	38.2 ± 4.8	41.3 ± 2.0
	Disintegration time (min)	<3	<3	<3
	Friability (%)	<1	<1	<1
	Water content (%)	3.00 ± 0.12	3.22 ± 0.65	3.41 ± 0.29
	Drug content (%)	101.05 ± 0.57	100.69 ± 1.28	99.65 ± 1.56

**Table 6:** Comparative mean (SD) of pharmacokinetic parameters of albendazole sulfoxide in rats after oral administration.

Pharmacokinetic Parameters	Albendazole Sulfoxide			Relative Bioavailability (%)
	Self-Microemulsion Tablets	Commercial Tablets	Powder	
AUC_0–∞_ (ng h/mL)	1982 ± 785	1502 ± 357	1591 ± 482	131.2 *
*C*_max_ (ng/mL)	3460 ± 235	1875 ± 123	1911 ± 198	
*T*_max_ (h)	1.00 ± 0.21	1.00 ± 0.16	1.00 ± 0.43	
*t*_1/2_ (h)	3.11 ± 1.21	3.02 ± 0.86	2.95 ± 1.02	

* Relative bioavailability with respect to commercial tablets (*p* ≤ 0.05).
